# An Association between Emotional Responsiveness and Smoking Behavior

**DOI:** 10.1155/2013/276024

**Published:** 2012-12-13

**Authors:** Robert D. Keeley, Margaret Driscoll

**Affiliations:** ^1^Division of Community Health Services, Denver Health Medical Center, Denver, CO 80204, USA; ^2^Department of Family Medicine, University of Colorado at Denver Health Sciences Center, Aurora, CO 80045-0508, USA; ^3^Driscoll Consulting, 866 Paragon Dr., Boulder, CO 80303, USA

## Abstract

*Introduction*. Emotional responsiveness (ER) has been theorized to play a protective role in pathways to tobacco initiation, regular use, and dependence, yet a possible association between ER and smoking behavior has not been studied. Our aim was to test whether measuring ER to a neutral stimulus was associated with decreased odds of current smoking. *Methods*. We measured ER and smoking status (current, former, and never) in two datasets: a cross-sectional dataset of persons with diabetes (*n* = 127) and a prospective dataset of depressed patients (*n* = 107) from an urban primary care system. Because there were few former smokers in the datasets, smoking status was dichotomized (current versus former/never) and measured at baseline (cross-sectional dataset) or at 36 weeks after-baseline (prospective dataset). ER was ascertained with response to a neutral facial expression (any ER versus none). *Results*. Compared to their nonresponsive counterparts, adjusted odds of current smoking were lower among participants endorsing emotional responsiveness in both the cross-sectional and prospective datasets (ORs = .29 and .32, *P*'s <.02, resp.). *Discussion*. ER may be protective against current smoking behavior. Further research investigating the association between ER and decreased smoking may hold potential to inform treatment approaches to improve smoking prevalence.

## 1. Introduction

In the United States about 20% of persons aged 16 and older report smoking, and smoking rates are higher among persons from lower socioeconomic strata [1]. While a range of treatments exist to help smokers quit, among persons from lower socioeconomic groups the evidence that interventions increase cessation is sparse [2]. Elucidation of novel factors associated with smoking behavior holds potential to substantively improve understanding of who experiments with tobacco, who becomes a regular user, or who successfully quits. Moreover, uncovering such person-level factors may inform adjustments to treatment approaches that prevent initiation, decrease prevalence of regular tobacco use, and support cessation. 

Person-level characteristics associated with smoking behavior are generally classified as psychopathology, personality, or gene related. Depressive symptoms, anxiety, psychosis, anger, social alienation, impulsivity, sensation seeking tendency, and attentional dysfunction have all been associated with current smoking [3]. Neurotic, extraverted, and open personality characteristics are associated with lifetime tobacco use [4]. Genetic variations in the nicotinic and dopamine receptors have been associated weakly with variation in nicotine dependence [5, 6]. Yet the generally weak associations between psychological states, personality traits, or receptor gene variations and smoking phenotype [7] raise some questions about clinical significance [8, 9].

Even small improvements in understanding pathways to regular smoking and dependence would hold potential to positively impact outcomes at the population level. The incremental PRIME model of addiction, which compiles extant demonstrated and proposed pathways to smoking phenotype, posits potentially important novel factors, perception, and response, as components of a hierarchical model of tobacco addiction [10]. Unfortunately, perception and response are conceptualized in PRIME as events occurring under specific circumstances that may be difficult to measure. Moreover, response has not been studied to our knowledge as a correlate of smoking behavior. To address these limitations, we have begun developing a general measure of an individual's tendency toward emotional responsiveness and have demonstrated evidence of a possible association between responsiveness and two other outcomes, blunted adherence to antidepressant adherence and decreased coronary heart disease risk [11, 12].

Evolutionary biologists have established evidence that emotional responses evolved to guide behavioral responses to an array of environmental challenges, thus maximizing the individual's chance of meeting survival-related goals [13, 14]. In addition, it is theorized that some mental health and possibly substance abuse disorders may be viewed through the lens of evolution and may in some cases provide short-term survival benefit to the afflicted person. For instance, nicotine in cigarettes may function to provide enhanced emotional responsiveness for individuals who are otherwise less responsive emotionally than their nonsmoking counterparts, thereby possibly enhancing social function and some short-term fitness. 

Measuring emotional responsiveness to relatively neutral stimuli is a practical approach to beginning to ascertain an individual's tendency toward emotional responsiveness [11]. Persons manifesting greater emotional responsiveness to relatively neutral stimuli may have a lower threshold to respond emotionally to a range of environmental stimuli, for example, public health admonishments against smoking, than their less responsive counterparts, and to exhibit subsequent behaviors protective against tobacco initiation and addiction. 

Thus, we examined how demonstrating a low threshold for responsiveness, ascertained by self-report of emotional responsiveness to a neutral facial expression (*ER-NFE*) [15], was associated with current smoking. We hypothesized ER-NFE would be associated with decreased odds of current smoking in nonoverlapping cross-sectional and prospective datasets. 

## 2. Method 

### 2.1. Sample and Study Design

Study participants were persons with diabetes attending seven primary care clinics at an urban community health system in the midwestern United States. The cross-sectional sample of persons with diabetes was recruited in waiting rooms, and by telephone from a diabetic registry. Baseline data were collected between May 2008 and March 2009. Subjects for the prospective dataset were screened by telephone for probable depression 2 days prior to a primary care visit, and those screening positive were recruited from the waiting room. Baseline data were collected from April 2009 to October 2011. Patients who were less than 18 years of age, were not English-speaking, were pregnant or breastfeeding, did not have a smoking status noted currently or within the previous 3 months, or were not able to answer survey questions requiring 30-day recall were excluded. In the prospective dataset, those not having major depressive disorder, or with bipolar disorder by diagnostic schedule, were disqualified. Both studies were approved by the Colorado Multiple Institutional Review Board (*COMIRB protocols no. 07-1180 and no. 08-1282*).

### 2.2. Outcome

#### 2.2.1. Smoking Behavior

For the cross-sectional dataset, self-reported smoking status was assessed at baseline from the electronic medical record, which categorized tobacco use as “current,” “former,” or “never.” We dichotomized smoking behavior as “current” versus “former” or “never.” In the prospective dataset, current smoking status (*yes/no*) was collected at 36 weeks by self-report. Self-reported smoking status is reliable and valid [16].

### 2.3. Independent Variables

#### 2.3.1. Factor of Interest: Emotional Responsiveness

Emotional responsiveness was ascertained using grey-scaled normalized Ekman neutral facial expressions (*NFEs*) [15]. We assessed response by asking: “What emotion best describes how you feel when viewing this picture?” Likert anchors were “fear,” disgust,” “anger,” “sadness,” “surprise,” “happiness,” or “no emotion.” For the cross-sectional study, a female NFE monograph, no. C2-3, was presented, and for the prospective dataset the no. C2-3 monograph and a second male NFE monograph, no. EM2-4, were also rated. For analytical purposes ER was dichotomized as any versus no response to the NFE(s) [14].

ER was associated with medication taking in a previous study, demonstrating face validity regarding associations with health-related behavior [11]. Discriminant validity appears good, as assessment of emotional responsiveness was not associated with educational attainment (*Pearson r* = .02), depressive symptom severity (*r* = .09), age (*r* = .08), gender (*r* = −.05), or race/ethnicity (*non-Hispanic black *(*r* = .13), *non-Hispanic white* (*r* = −.09), *and Hispanic* (*r* = −.05) *race/ethnicity*) in the prospective dataset. No significant associations between emotional responsiveness and personality traits identified by the 5-factor model were noted (*r*'s < .15) [17], and 3-month test-retest reliability was good at .79. 

**Figure 1:**
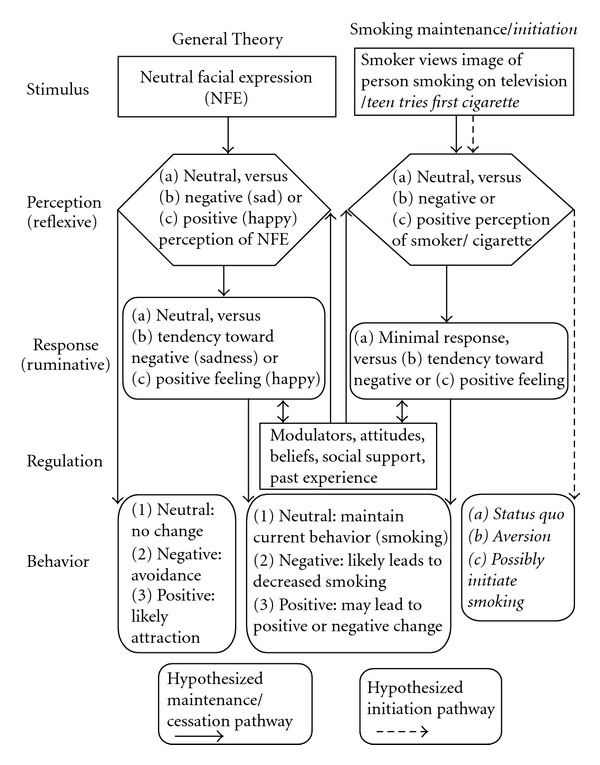
Theory of emotional perception, response, and health-promoting behavior applied to tobacco use.

 As a theoretical framework for this study, we synthesized theories from Paul Ekman, Mary Phillips, Richard Nesse, and others developed over the last 4 decades. In the 1970s Ekman described how perception of a neutral facial expression varied, with substantial numbers of persons rating the face as revealing negative or positive emotion. Neurobiological research uncovered pathways from general perception of the environment to emotional responsiveness and then to subsequent behaviors. In fact, evolutionary biologists have established evidence that emotions evolved to guide behavioral responses to an array of perceived environmental challenges, thus maximizing the individual's chance of meeting survival-related goals [18]. In perceiving and judging their environment, persons will often have a measurable emotional responsiveness that drives subsequent behavior. According to this resultant theory of emotional perception, response, and health-promoting behavior (Figure 1), we theorized that persistent smoking behavior would be more prevalent among persons with relatively blunted emotional responsiveness when presented with neutral stimuli. 

Moreover, sensitivity toward perceiving emotion varies by mood state and personality [19]. Neutrally valenced expressions may tend to elicit less ER than positively or negatively valenced facial expressions [20]. Thus, perception of and emotional responsiveness to a NFE may represent a simple way to ascertain subtle differences in activation thresholds for ER [11, 21]. Persons exhibiting emotional responsiveness to a neutral facial expression may more likely respond emotionally to a range of environmental stimuli, for example, public health admonishments against smoking, than their less responsive counterparts. Consistent with this theoretical framework, we theorized that emotional nonresponsiveness to NFE would be associated with increased smoking behavior, while emotional responsiveness to NFE would be associated with smoking abstinence.

#### 2.3.2. Possible Confounders

Based upon literature review and theoretical plausibility, possible confounders of an association between ER and current smoking were selected and determined at baseline from electronic medical record review, pharmacy refill records, and self-report (Table 1).

*Demographic factors *included age, educational attainment, race/ethnicity (Hispanic, non-Hispanic (NH) White, and NH Black), gender, unadjusted income, and insurance status. Educational attainment was dichotomized as less than high school versus a high school equivalent education or higher. Insurance was categorized as public, including Medicare and Medicaid, a partial coverage program for otherwise uninsured indigent persons, or private.

*Psychosocial variables* were measured through a series of survey questions. A collaborative relationship with the primary care clinician was assessed with a 3-item scale from the Helping Alliance Questionnaire [22].

Self-efficacy [23] was ascertained with the first question from the General Self-Efficacy scale. A single question assessment has been used before in tobacco studies [24].

Social support was assessed by self-report of number of household members, and social function with a question from the Short-Form 36 instrument (*SF-36*) assessing the extent to which mental or physical health problems affect social activities [25].

*Comorbid measures *included a measure of probable depressive disorder as measured by the Patient Health Questionnaire-2 (*PHQ-2*) [26]. A score of 3 or higher was considered to represent probable major depressive disorder. Depression often precedes smoking initiation and experimentation [27]. Lifetime generalized anxiety disorder was assessed with a diagnostic schedule.

*Functional assessments* were also included in the study. Smoking is associated with chronic pain [28], and bodily pain was determined from the sum of two questions from the SF-36 [25]. Body mass index (BMI) was measured both as a continuous variable and as a categorical variable (*per National Heart Lung and Blood Institute criteria*) [29].

Because visiting a primary care office may increase the odds of receiving a smoking intervention, the number of primary care visits over the previous 12 months at the urban health care system was ascertained from the EMR. Current narcotic use has been associated with smoking and was defined as filling a monthly prescription for narcotics at least two times in the previous 4 months as determined from automated pharmacy refill records. 

### 2.4. Analytical Approach

For both cross-sectional and prospective models, we assessed for univariate associations between the factor of interest, possible confounders, and current smoking. We selected those variables associated at *P* < .10 (*Spearman's correlation*) for further analysis. We conducted a multivariate logistic regression analysis with the dependent variable current smoking and the independent variable ER, entered possible confounders in blocks by domain (*demographic, psychosocial, comorbid, etc.),* and retained those variables associated with smoking at *P* < .10 for the final model.

#### 2.4.1. Size of the Clinical Association

For dichotomous risk factors, nonparametric methodology generates the “area under the curve” (AUC) size of the clinical association (ES). An AUC = .56 is equivalent to a small association (*Cohen's* *d* = .2), while AUC = .64 and AUC = .71 are equivalent to medium and large associations (*Cohen's* *d* = .5  *and .8, resp.)* [30].

We used SAS 9.2 (SAS, Cary, NC) with PROC SURVEYMEANS and SURVEYLOGISTIC nested by clinic site for descriptive and multivariate analyses. Alpha was set at *P* < .05 for multivariate analyses.

## 3. Results

### 3.1. Characteristics of the Participants (Table 1)

For the cross-sectional dataset, we recruited 129 persons with diabetes for inclusion in the study. Of these, 127 with complete information regarding the primary outcome smoking status were included in the current study. For the prospective dataset, we included 107 subjects with available 36-week smoking status.

A relatively high proportion of the cross-sectional sample reported current smoking (52/127, 40.9%). About half reported never smoking (63/127, 49.6%), and 9.5% (12/127) were former smokers. In the prospective dataset, 34.6% (37/107) were current smokers.

### 3.2. Hypothesized Correlate: Emotional Responsiveness

In the cross-sectional dataset we found that 36.2% (46/127) of the subjects endorsed a non-neutral ER-NFE (e.g., sadness, fear, anger, happiness, and surprise). In the prospective dataset, 70.1% (75/107) gave a non-neutral response to at least one of two NFEs. The rate was higher in the prospective dataset because the respondent only had to provide an emotional response to one of two NFEs, while in the cross-sectional dataset only one NFE was rated.

In the cross-sectional dataset, over twice as many non-smokers (46.7%  (35/75)), including never and former smokers, reported emotional responsiveness as current smokers (21.2% (11/52)). In this dataset, almost half of never smokers endorsed emotional responsiveness to the neutral facial expression (49.2%  (31/63)), in contrast with former smokers of whom only 33.3% (4/12) appeared responsive. Overall, current smokers were significantly less likely than never smokers to report ER-NFE (*Chi-square *= 8.2, *P* = .004). Former smokers did not demonstrate significantly different rates of ER-NFE than current (*Chi-square *= .81, *P* = .47) or never smokers (*Fisher's Exact test P* = .36).

In the prospective dataset, 77.1% (54/70) of non-smokers reported ER-NFE, compared with 56.8% of current smokers (21/37).

### 3.3. Multivariate Logistic Regression Analyses (Table 2)

#### 3.3.1. Cross-Sectional

 The adjusted odds for smoking were over three times lower for patients reporting ER-NFE relative to their less responsive counterparts (OR = .29, 95%  CI  .11, .72,  *P* = .008). The multivariate model was adjusted for age, current narcotic medication use, and self-efficacy. Increasing age was associated with lower odds of smoking in this cohort (OR = .57, 95%  CI  .36,.89,  *P* = .014*, for 1 SD (10.8 years) increase from median age (50.0 years))*. Self-efficacy was associated with lower probability of current smoking (OR = .17, 95% CI .05, .66, *P* = .01). Current use of narcotic pain medication appeared to be associated with increased odds of smoking (OR = 2.50, 95% CI 1.08, 5.81, *P* = .033). The model  *c*-statistic was .76.

#### 3.3.2. Prospective

Patients endorsing ER-NFE were over 3 times less likely to smoke than their nonresponsive counterparts (OR = .32, 95% CI .13, .82, *P* = .018). The multivariate model was adjusted for educational level and BMI. The  *c*-statistic was .71. 

### 3.4. Clinical Association between ER-NFE and Current Smoking

In the cross-sectional and prospective datasets, the AUC clinical associations for ER-NFE comparing current to non-smokers were .63 and .61, respectively. These would be considered small associations equivalent to Cohen's  *d*'s of .43 and .41. The size of the association was medium when comparing current to never smokers in the cross-sectional dataset (AUC ES = .64, Cohen's *d* = .51).

## 4. Discussion

This is the first study of which we are aware to demonstrate that a measure of emotional responsiveness to neutral facial expression was robustly associated with not smoking in two nonoverlapping samples of urban-dwelling primary care patients. In adjusted analyses, current smoking was over 3 times less likely for persons endorsing emotional responsiveness relative to their nonresponsive counterparts. The extent of the clinical association was more than twice a recommended threshold for association between independent factors and current smoking, demonstrating a robust level of clinical significance [9, 31]. Emotional responsiveness was not related to a range of potential confounders including depressive symptoms, general anxiety disorder, and personality domains. Thus, our findings signify that the measure of emotional responsiveness appears to represent a novel, positive correlate of tobacco use behavior.

Other potentially modifiable correlates of smoking behavior in the cross-sectional dataset included self-efficacy and narcotic use. Self-efficacy appeared protective against current smoking, and previous studies have suggested possible associations between self-efficacy and decreased current smoking [2]. Narcotic use has been positively associated with current smoking [32]. In one study, persons receiving prescription narcotic pain medication were over twice as likely to smoke as their peers who did not receive narcotic pain relievers. On the one hand, tobacco users are more likely to develop chronic pain than persons who do not smoke, thereby increasing likelihood of narcotic treatment for pain. On the other hand, the finding may be explained in part because tendency toward addiction to one substance (e.g., tobacco) has been associated with addictions to other substances (e.g., narcotic medications). 

In the prospective multivariate analysis of patients with probable depression, greater than 12 years education was associated with decreased odds of smoking, consistent with previous research [33], while increasing BMI was associated with higher odds of smoking. The latter observation is not surprising, as both tobacco use and obesity are more prevalent among depressed persons, relative to their nondepressed counterparts [29]. The samples had high rates of smoking, likely because they were drawn from patients of mostly low socioeconomic status attending an urban public health care system.

### 4.1. Strengths and Limitations

Strengths of the study include the theory-driven hypothesis, the adjustment for a broad set of possible confounders, and confirmation of the cross-sectional findings in a second prospective analysis in a nonoverlapping dataset. It is possible that the association between a neutral response to NFEs and current smoking was explained by a nicotine-related influence; that is, nicotine may suppress emotional responsiveness. However, this explanation is unlikely because former and current smokers in the cross-sectional study had lower rates of ER-NFE than never smokers. Threats to external validity include two relatively small samples of lower SES individuals from one health system. Validity would be improved by demonstrating associations between ER-NFE and other measures of emotional responsiveness such as electrophysiological correlates of facial expression processing [20].

## 5. Conclusion

The study appears to demonstrate a robust association between emotional responsiveness to neutral facial expression and decreased odds of current smoking. As even modestly improved understanding of tobacco use holds potential to inform improvements to prevention and treatment approaches, we recommend further research to confirm and further explore this initial description of a relationship between ER-NFE and current smoking in other study populations.

## Figures and Tables

**Table 1:** Study populations.

Characteristic	*n *(cross-sectional dataset)	Mean (95% CI; range) or frequencies	*n *(prospective dataset)	Mean (95% CI; range) or frequencies
Demographic				
Age (years)	127	51.3 (49.5–53.2; 26–77)	107	50.9 (48.4–53.5; 18–66)
Gender	127	52.0% female	107	71.0% female
Race-ethnicity	127	40.2% NH White 26.8% NH Black 33.1% Hispanic	107	21.5% NH White 32.7% NH Black 35.5% Hispanic 2.8% NH Native American 7.5% NH other
Educational attainment (years)		NA	100	24.0% < high school 76.0% high school or greater
Income (US dollars)	115	$10,309; (7598.5–12,919.2; $0–$40,086)		NA
Insurance	127	51.2% no insurance 48.8% public (Medicaid or Medicare) or private		NA
Smoking status	127	40.9% current 9.5% former 49.6% never	107	34.6% current 65.4% former/never
Psychosocial				
Collaborative patient-clinician relationship	125	15.6 or “moderately good” (15.2, 16.0; 4–18)		NA
Self-efficacy	123	3.2 or “good” (3.0, 3.4; 1–4)		NA
Social				
Health interference	122	2.8 or “moderate interference” (2.5, 3.0; 1–5)		NA
No. of household members	127	1.5 (1.4, 1.7; 1–6)		NA
Comorbid				
Probable depressive disorder (PHQ-2 ≥ 3: or PHQ-9 depressive symptoms score^†^)	127	31.7%	107	16.0^†^ (15.2, 16.8; 10–23)
Generalized anxiety disorder		NA	107	24.2%
No. of physical comorbidities (0–10)	107	NA		2.6 (2.2–2.9; 0–7)
Functional				
Bodily pain	124	6.8 or “moderate” (6.3, 7.4; 2–13)		NA
Body mass index	126	0.8% underweight 12.7% normal 23.0% overweight 63.5% obese	107	0% underweight 18.7% normal 26.2% overweight 55.1% obese
Medical care				
No. of primary care visits/previous year	126	4.9 (4.4, 5.5; 0–16)		NA
Current narcotic use	126	35.7%		NA
Factor of interest				
Emotional response to neutral facial expression(s)^§^	127	36.2%	107	70.1%^§^

^§^Any emotional response to one of two neutral Ekman monographs; NH: non-Hispanic; ^†^PHQ: Patient Health Questionnaire-9 depressive symptoms score (range 0–27).

**Table 2:** Multivariate logistic regression models.

	Estimate (SE)	Adjusted odds ratio (95% CI)	Pr (>|*t*|)
(A) *Cross-sectional dataset* (*n* = 122)°			
Dependent variable = current			
smoking at baseline (*n* = 49)			
Intercept	.83 (.56)		.13
Self-efficacy	−1.75 (.68)	.17* (.05, .66)	.01
Narcotic pain medication use	.46 (.22)	2.50 (1.08, 5.81)	.033
Emotional responsiveness	−.62 (.24)	.29 (.11, .72)	.008
Age	−.56 (.23)	.57* (.36, .89)	.014
*c*-statistic = .76			

(B) *Prospective dataset* (*n* = 100^*Ω*^)			
Outcome = current smoking at 36			
weeks after baseline (*n* = 44)			
Intercept	−1.02 (.74)		.17
At least 12 years education	−.56 (.26)	.33 (.12, .90)	.03
Emotional responsiveness	−.57 (.24)	.32 (.13, .82)	.018
Categorical body mass index	.54 (.29)	1.72 (.98, 3.03)	.058
*c*-statistic = .71			

°*n* = 5 with missing educational attainment data; *odds of smoking for each 1 SD increase from median score.

^*Ω*^ *n* = 7 with missing self-efficacy (4) or use of narcotic pain medication (1) data.
